# On the Premature Dementia of Puberty and Adolescence

**Published:** 1903-06-06

**Authors:** W. Lloyd Andriezen

**Affiliations:** late Deputy Medical Superintendent Darenth Asylum; formerly Medical Officer and Pathologist at the West Riding Asylum


### HOSPITAL CLINICS AND MEDICAL PROGRESS.

### ON THE PREMATURE DEMENTIA OF PUBERTY AND ADOLESCENCE.

(*Concluded from page* 150.)

By W. LLOYD ANDRIEZEN, M.D.Lond., late Deputy Medical Superintendent Darenth Asylum; formerly Medical Officer and Pathologist at the West Riding Asylum.

The five varieties of dementia præcox may now be passed in review *seriatim.*

(*a*) The simple or non-confusional form (*D. p. simplex*). This is the fundamental and essential form of dementia præcox, pure and simple. It is characterised by mental enfeeblement and apathy beginning in puberty or adolescence, progressing slowly and uneventfully, and passing into a moderate but permanent degree of mental enfeeblement (dementia) which, however, need not incapacitate the individual for the simpler duties of work and social life. Morality is weak and chastity frail in such individuals, but active criminal or anti-social instincts are not prevalent in this class. Unless their previous history is known, it is hardly possible to distinguish these cases from imbecility or congenital weak-mindedness.

(*b*) The confusional or hebephrenic form (*D. p. hebephrenica*). On the basis of an incipient and slowly progressive mental enfeeblement such as characterises dementia præcox simplex, there is grafted a state of confusional insanity, the *tout ensemble* of which constitutes hebephrenia. Female patients, and occasionally also male patients, of this class frequently exhibit signs and symptoms of an "hysterical" character. Cases of hebephrenia exhibit a medley of confused mental states—excitement, depression, and hallucinations. The hallucinations are about as frequently visual as auditory, but they are of a mobile, transient and fleeting character, and the mood of the patient is correspondingly variable. Depression of spirits, when present, is not of the nature of a true melancholia. The patient displays attitudes and postures of a dramatic, ecstatic and histrionic character, he utters phrases and exhortations after the manner of an orator, and indulges in silly laughter, in grimaces, and occasionally in tears. The mental confusion or delirium is sometimes liable to take on a more acute or severe form, in which case the general condition of the patient is one of stupor—a waking but dream-like delirium in which consciousness is impaired, the prevailing condition being one of subconscious automatism. Occasionally this condition is interrupted by a sudden and transient outbreak of violence. The physical condition is one of feeble circulation in the extremities, and of increased muscular irritability (myoidema) with exaggeration of the knee-jerks. The following brief record of an illustrative case is appended :—

J. R. S——, male, aged 19 years, single, began to develop symptoms of mental disorder in his 19th year. The symptoms consisted of mental confusion, refusal of food, restlessness and resistiveness to everything required to be done. He would also walk about half-dressed and indecently expose himself. On attempting to dress or undress him he would get violent. The flesh showed a tendency to develop bruises readily, the temperature was 99·2°, and there were sordes on the lips. He preserved a stubborn silence (mutism). Two years previously, at the age of 17 years, he had suffered from attacks of sleeplessness and general apathy. His present condition was preceded by some weeks of alternate excitement and stupor (mental confusion) attended with hallucinations and eventually with delusions of poisoning, so that he refused to take food for some days. The general attitude is one of restless, watchful suspicion—a persecutory mood seems to be dominant—and of resistiveness to everything (to dressing and undressing, to feeding by spoon and feeding-tube, and to the catheter). The pupils are dilated, the bowels constipated, and at times he wets himself. The diagnosis was dementia præcox of the hebephrenic variety, tending to stupor. In course of time he improved under treatment, the mental confusion, persecutory mood, delusions, resistiveness, mutism, and degraded habits passed away, leaving a permanent residue of mental enfeeblement (dullness and slowness of mind). He has gained flesh in the process of improvement, and can do a little easy work.

(*c*) The catatonic form (*D. p. catatonica*).—In this variety of insanity the degree of cerebral weakening is greater generally, the clouding of consciousness is more profound, and a peculiar state of stupor with cataleptiform rigidity and resistance to all foreign interference (negativism) is manifested. A further development of this phase of negativism is seen in refusal of food, retention of urine, and mutism or stubborn silence occasionally interrupted by the utterance and repetition of meaningless sounds and syllables (verbigeration). The classical picture of this form of insanity was drawn by Kahlbaum more than twenty-five years ago. He had described "the bent body, the stiffened arms and legs, the wrinkled brow and downcast eyes, the stubborn silence, the negativism, the vaso-motor disturbances, the stereotyped way of doing things, the verbigeration, the isolated convulsions, and the unforeseen sudden impulses to violence" which the patient exhibits. The mental processes in catatonia may find expression in a monotonous flow of words and phrases often repeated, while the manner is both dramatic and pathetic. Bodily restlessness is not marked. Indeed, as Kahlbaum pointed out, catatonic patients are of a "soft temperament, quiet and gentle," though they seem to be haunted by vague feelings of persecution. The sexes are unequally affected by this form of dementia præcox, females predominating in number, and its onset occurs in most cases towards the close of adolescence, though a few occur at puberty. Some interesting pathological parallels between catatonia and family myoclonia have been drawn by Scandinavian observers (Svenson, Lundborg and Faber). General cutaneous sensibility is diminished, the tendon-reflexes are exaggerated, retention of urine and fæces occurs intermittently, cyanosis and coldness of the extremities and some œdema of the limbs (without cardiac or renal mischief) are met with, and dermographism of varying degrees is almost constantly present.

(*d*) The paranoid form (*D. p. paranoides*). This variety of dementia præcox is rare. It is marked by steady and progressive intellectual enfeeblement, in the course of which abundant hallucinations and delusions are evolved. The delusions may last for years, in spite of the steady mental deterioration or dementia which occurs. The mental atmosphere is dull and clouded, there is complete absence of the lucidity and mental keenness of paranoia, and the delusions never develop and cohere into a systematic and logical whole. They are exuberant in number and in character, but undergo no linkage with the past life of the patient. Hence, also, there is not produced the gradual transformation of the ego characteristic of paranoia. The termination in all cases is one of marked dementia.

(*e*) The criminal form (*D. p. criminalis*). In this variety of dementia præcox the onset of mental deterioration at puberty is complicated by the development of strong criminal instincts, sexual, homicidal, and destructive (especially arson). These impulses seem from time to time to manifest themselves in the patient with irresistible force (periodic exacerbations). Patients of this class are apt to be confounded with the "moral imbecile" whose anti-social or criminal instincts are due mainly to congenital defect, and it is only by a knowledge of the patient's past history that we are enabled to differentiate the two conditions. Similarly, simple dementia præcox must be differentiated from hysteria, neurasthenia, and congenital imbecility or weak-mindedness. The hebephrenic variety should not be confounded with mania, melancholia, or recurrent and circular insanity, the catatonic form should be distinguished from vesanic stupor and catalepsy on the one hand and from general paralysis on the other, while paranoid dementia should be separated from the "chronic delusional insanity of systematic evolution" of Magnan.

Before concluding attention may be drawn to certain interesting parallelisms between dementia præcox and hysteria major, an allied psychosis. First, in the anæsthesias of hysteria major we find a parallelism with the incapacity for attention (apathy) and for will (aboulia) of early and mild dementia præcox. Secondly, in the paralyses of hysteria major we find an analogy with the (persecutory and hypochondriacal) delusions of dementia præcox, and like the paralyses such delusions are of fleeting and ephemeral nature, though a few eventually tend to get stereotyped. Thirdly, in the paralytic contractures (stereotyping of paralyses) of hysteria major we find a parallel with the catatonic and stereotyped attitudes and acts present in the graver forms of dementia præcox.

From the foregoing remarks it will thus be evident that dementia præcox comprises a group of insanities peculiar to the period of puberty and adolescence, that it is essentially a slowly progressive dementia on which may be grafted states of mental confusion and dream-like delirium (a foundation of dementia and a superstructure of confusional insanity), that it is not psychologically a mania or a melancholia, that it is a form of mental degeneration presenting curious analogies to hysteria major, and that it is worthy of occupying a distinctive place in the scheme of mental diseases.

